# Belief beyond reason: a radical relativist hinge epistemology

**DOI:** 10.1007/s44204-025-00244-4

**Published:** 2025-01-31

**Authors:** Drew Johnson

**Affiliations:** Austin, USA

**Keywords:** Hinge epistemology, Epistemic relativism, Non-evidentialism, Skepticism, Epistemic injustice, Deep disagreement

## Abstract

Hinge epistemology is sometimes thought to have controversial relativist and non-evidentialist commitments. This paper develops and motivates an explicitly relativist and radically non-evidentialist version of hinge epistemology, following and combining aspects of Ashton’s (2019) defense of relativist hinge epistemology and Pritchard’s (2016) defense of a non-epistemic reading of hinge commitments. I argue that radical relativist hinge epistemology shares in a main attraction of hinge epistemology in general, namely, offering a dissolution of closure-based radical skeptical problems. I then motivate RR as a *social hinge epistemology* by showing that it is particularly well-suited for fruitful applications in topics such as deep disagreement, testimonial injustice, and hermeneutic injustice.

Let us not pretend to doubt in philosophy what we do not doubt in our hearts.

–Charles Sanders Peirce, *Collected Papers Vol. V *(1934), §265

So I am trying to say something that sounds like pragmatism. Here I am being thwarted by a kind of *Weltanschauung*.

–Ludwig Wittgenstein, *On Certainty* (1969), §422

## Introduction

Hinge epistemology, a branch of epistemology informed by Wittgenstein’s *On Certainty *(1969) (henceforth *OC*), maintains that rational evaluation in general is possible only because of certain commitments (“hinges”) that are legitimately held though evidentially unsupported. The goal of this paper is to develop and motivate an under-explored option for hinge epistemology. This view, which I shall label “Radical Relativist Hinge Epistemology” (RR), combines an epistemic relativist approach with a non-evidentialist account of hinges’ role in epistemic evaluation. Whereas others have sought to weaken or avoid the apparent relativist and non-evidentialist leanings of hinge epistemology (Neta, 2019; Piedrahita, 2021), I argue that when properly employed, these are not consequences to be feared but rather provide hinge epistemology with useful tools for analyzing certain phenomena in social epistemology.

The overall argumentative aims of this paper are in a sense modest and ecumenical. The aim is to provide a “proof-of-concept” for a relativist and non-evidentialist approach and then to show some of the explanatory payoffs of this approach. The aim is not to provide a knock-down argument that this is the “one true” way to develop hinge epistemology that would avoid insurmountable problems for all other approaches. Given that “showing that a view entails relativism is almost always considered tantamount to showing that it is untenable” (Ashton, 2019b, 587), arguing for the tenability of a relativist hinge epistemology in itself is an important contribution to discussions of hinge epistemology. Likewise, while hinge epistemology has non-evidentialist tendencies, non-evidentialism remains a controversial view (see Moretti & Pedersen, 2021 for recent discussion). I argue that a firmly non-evidentialist hinge epistemology is, despite initial appearances, able to address perennial skeptical problems.

The structure of the paper is as follows: In the remainder of this section, I provide a general overview of hinge epistemology and introduce RR. In Sect. 2, I present an argument for RR based on a plausible rational grounding principle. I also show how RR can dissolve skeptical problems in a manner characteristic of hinge epistemology. In Sect. 3, I apply RR to some topics in social epistemology and argue that RR is particularly well-placed to explain the epistemology of deep disagreement and certain forms of testimonial and hermeneutical injustice. Overall, the paper motivates RR as a viable approach in hinge epistemology with an explanatory payoff in social epistemology; RR can thus be considered a form of *social hinge epistemology.*

### Introducing radical relativist hinge epistemology

Hinge epistemology comprises a family of views about the structure of epistemic warrant, typically motivated by providing a distinctive dissolution of radical skeptical problems. Though differing substantially in their details, all hinge epistemologies share in the following commitments (Ashton, 2019a):

1. *Lack of evidential support*: Certain commitments that are central to epistemic practice lack evidential support, in some sense.
2. *Legitimacy*: Nevertheless, these commitments are legitimate to hold, in some sense.

In *OC*, apparent examples of hinges are often mundane empirical claims. Some instances are claims such as “The earth has existed for many years” (*OC* §411), “Houses don’t suddenly turn to steam” (*OC* §513), “My friend hasn’t got sawdust in his head” (*OC* §281), and, most famously, the Moorean claim that “I have hands.” There is disagreement however over what counts as a good instance of a hinge, in light of the seemingly counterintuitiveness of claiming that we could not know or have evidential support even for such a claim as that one has hands. For instance, Annalisa Coliva, in her development of Wittgenstein’s ideas, argues that we should not think of “I have hands” as a hinge, but rather only very general empirical claims, such as that the earth is many years old, can count (2015). Duncan Pritchard takes an even more restrictive approach in a sense, by maintaining that really there is only one hinge commitment (albeit one that can be manifested or codified in other more specific claims)—a commitment to the proposition that one is not radically and fundamentally in error (2016).

I will take a permissive approach to hinges and argue that there are no in-principle restrictions on the possible contents of hinge commitments; any proposition could count as a hinge (and hence lack evidential support) for some epistemic perspective, including the claim that one has hands, that one’s name is such-and-such, that houses do not turn to steam, and so on. I defend this permissive approach by arguing that what matters for determining whether something is a hinge is the role that it plays in an individual’s epistemic perspective and that the content of the hinge is not essential to this role. However, this can seem to have the counterintuitive result that many basic empirical claims could lack evidential support. It will be simplest to address this apparently counterintuitive result at the outset.

Here is a two-pronged strategy to address the worry that even basic empirical claims could lack evidential support. First, the claim that hinges lack evidential support I think should be understood as applying to *internalist* forms of epistemic support. The point is that we lack genuine reflectively accessible justification that we could cite as reasons for belief when it comes to hinges (although we may falsely believe we have such reasons). Yet we may still have knowledge of hinges in a sense, since we could have externalist forms of justification for them. Duncan Pritchard takes this sort of strategy, by framing his version of hinge epistemology in terms of rationally grounded knowledge, with the idea that although we lack rationally grounded knowledge of hinges, that does not preclude having knowledge of them in some other form (i.e., knowledge that is not rationally grounded) (2016). Crispin Wright likewise argues that we can have a form of epistemic support for hinges—epistemic *entitlement*—that does not include making any kind of cognitive achievement to come to have evidence for belief (2004). That is, in his view, there can be epistemic warrant without evidential support.

Even with this distinction in kinds of epistemic justification in mind, the claim that hinges lack evidential support can still seem counterintuitive. Far from being claims about which one can have *no* (internalist) evidential support, the examples of hinges we find in Wittgenstein and other writers initially seem to be things about which we would tend to think of ourselves as having *the most* evidential support possible. What could I have more justification for believing than that I have hands? Here, the second prong of our strategy comes into play and reveals the core insight of hinge epistemology. We can be mistaken about where we have evidential support for belief—we might think we have genuine evidential support when we do not. In the case of our most certain commitments—our hinges—what we might think of as providing strong epistemic support in fact is not able to do so, because of the special role that hinges play in our epistemic systems. In Sect. 2, I use a plausible rational grounding principle that I think we find at work in *On Certainty* and in some developments of Wittgenstein’s ideas in the literature that explains why we could be mistaken in this way about what we have evidential support for believing.

### Hinge epistemology

Hinge epistemologies differ in the way they articulate the idea of a “hinge” and how hinges can be legitimate to hold without evidence. I will consider four prominent approaches taken by proponents in the literature, in the views of Coliva (2015), Moyal-Sharrock (2004), Pritchard (2016), and Wright (2004). Theories can be categorized according to whether they view hinges as (1) propositional (Coliva, Pritchard, Wright) versus non-propositional (Moyal-Sharrock); (2) doxastic (Wright, Coliva) versus non-doxastic (Pritchard, Moyal-Sharrock); (3) epistemic (Wright) versus non-epistemic (Coliva, Moyal-Sharrock, Pritchard); and (4) in the possible scope of the content of hinges, i.e., whether hinges are variable (and in what way) or invariant. According to RR, hinges are propositional, doxastic (in one sense of “doxastic”), non-epistemic, and variable in being fixed relative to individuals’ epistemic perspectives.

The propositionality question concerns whether hinges take propositions as their objects or whether they are best thought of as non-propositional rules. Arguably, Wittgenstein held a view of propositionality according to which in order for something to be a proposition, it had to be *descriptive* and possible to *falsify* (that is, it must satisfy bipolarity) (*OC* §95, §98, §404; see Coliva, 2010 for discussion). By contrast, norms, rules, and ways of life are not propositional, since they either are not descriptive (but rather prescriptive) or fail bipolarity. As embodied in ways of acting, they are not the sorts of things that can be assessed as true or false in confrontation with reality. Moyal-Sharrock develops a view on which hinges are rules that are embodied in ways of acting and hence are non-propositional (2004). While a hinge might be expressed linguistically, for instance, in the sentence “I have hands,” the hinge is itself embodied in ways of acting, such as through the unquestioned reliance on my hands to perform many activities.

This view on propositionality is not the only available option and may itself come with some significant costs. At the very least, it seems to entail a commitment to a controversial view in meta-ethics: so long as one thinks of ethical (or, more broadly, normative) claims as expressing non-cognitive states and encouraging certain ways of acting rather than as describing some realm of normative fact, the Wittgensteinian view of propositionality would seem to entail that ordinary ethical claims do not express propositions. There are well-known difficulties with this view—most famously, the Frege-Geach problem. The problem is that moral sentences can clearly figure in valid arguments and inferences with non-moral empirical premises, yet it is unclear how or whether the non-cognitive states they are supposed to express could inferentially integrate with the cognitive states expressed by non-moral sentences (Geach, 1960, 1965; Schroeder, 2010). Analogous problems arise for the non-propositional approach to hinges: The sentences which express hinges (such as “I have hands”) clearly seem capable of entering into semantic and logical relations with non-hinge sentences. Even in circumstances where “I have hands” expresses a hinge commitment, it should still remain true that that sentence, in that circumstance, nevertheless entails that one is not a (handless) brain-in-a-vat. Indeed, a central theme in discussions of hinge epistemology concerns the applicability of closure principles to hinges, i.e., whether and in what way knowledge or warrant might be closed under known entailment between hinges and non-hinges (Jope, 2019; Pritchard, 2016; Wang, 2020; Zhang, 2021). The Wittgensteinian view on propositionality however is incompatible with the clear semantic integration of sentences expressing hinges with other ordinary sentences.

How then are we to capture the characteristic differences between hinges and other ordinary empirical claims? An alternative is to acknowledge significant differences in kinds of content (e.g., empirical vs. normative), without locating this difference as a matter of propositionality. Without disputing that hinges could be rules, norms, or embodied in ways of acting, we can nevertheless maintain that they are propositional, using a more minimal account of propositionality. The differences between hinges and non-hinges are then located somewhere other than in their status as propositions. Following Pritchard, Coliva, and Wright, I will view hinges as propositions.^1^

Views that take hinges to be doxastic see them as akin to belief. But there are different ways to characterize belief, so it is important to be clear about what is meant in saying that hinges are beliefs (see Simion et al., 2021 for discussion). A minimal approach holds that belief is just a propositional attitude of firm commitment to the truth of its content. If nothing else, if a hinge commitment is a commitment to a proposition, it is at least firm commitment to the truth of that proposition. As long as hinges are admitted to be propositional, they will also count as doxastic in this minimal sense. A stronger view would hold that in addition to firm commitment to truth, a propositional attitude only counts as a belief if it is in addition *knowledge-apt*. This means (assuming a Justified-True-Belief model for knowledge) that only commitments which are capable of having some kind of epistemic justification could count as beliefs. This notion of knowledge-apt belief is neutral with respect to the kind of justification that counts—so, a belief could be knowledge-apt if it is capable of justification in either internalist or externalist terms. Given the above discussion of the possibility that hinges could enjoy non-internalist forms of epistemic warrant, the knowledge-apt belief view could also acknowledge hinges as doxastic (depending on whether and how one thinks hinges have an epistemic warrant). Finally, one might endorse a yet more restrictive notion of belief, that of *rationally grounded knowledge-apt belief* (Pritchard, 2016). Something only counts as rationally grounded knowledge-apt belief if it is a firm commitment to the truth of a proposition, where the commitment can be based on and is responsive to reasons.^2^

I use the minimal notion of belief as a criterion for determining whether hinges are doxastic. Doing so is useful because it separates the question of whether hinges are doxastic, or belief-like, from questions about the epistemic status of hinges as warranted or warrantable, which I take to be a separate question. The minimal account also avoids the somewhat awkward commitment of more robust views of belief, to the claim that one does not really *believe* hinges such as “I have hands,” “My friend does not have sawdust in his head,” and “The earth is many years old.” The denial that one can believe these things is quite revisionary of ordinary usage. Moreover, the minimal account can make sense of our attributions of beliefs to young infants and non-human animals, who might not be responsive to reasons in the way needed to have rationally grounded knowledge-apt beliefs.^3^ On the minimal account, RR (like some other versions of hinge epistemology) counts as a doxastic view. According to the knowledge-apt notion of belief, hinges also count as doxastic on RR, because RR is compatible with holding that there are externalist varieties of warrant available for hinges. For this reason, RR can again avoid an awkward commitment to the claim that one could not really *know* such things as “I have hands,” as discussed above. However, if we think of belief as rationally grounded knowledge-apt belief, RR counts as non-doxastic, since it denies that we can have genuine reflectively accessible reasons for belief in the truth of the hinge. So, although I prefer to consider RR as a doxastic view, this is only because I prefer a minimal account of doxastic states. RR agrees in substance with at least one account that is explicitly construed as non-doxastic—Pritchard’s—in holding that hinges are not rationally grounded knowledge-apt beliefs.

Epistemic approaches view hinges themselves as appropriate targets of epistemic evaluation or as having some positive epistemic status, albeit something different from evidential support. Moyal-Sharrock’s view is thoroughly non-epistemic, since hinges’ status as rules in her account means that they are not appropriate targets for epistemic evaluation in the first place. While Coliva views hinges as partly constitutive of rationality, she does not take this to provide a positive epistemic reason to accept hinges as true, and hence, her view is not an epistemic one. Pritchard views hinges as arational because they are neither supported by rational grounds nor can they provide support, and hence also provides a non-epistemic view. RR is a non-epistemic view in the same sense as Pritchard’s. Wright’s view by contrast is epistemic, because Wright regards hinges as enjoying positive epistemic warrant through enabling valuable cognitive projects that result in good epistemic results (see also Pedersen, 2021 on epistemic consequentialism as a foundation for entitlement theory).

Another dimension along which approaches to hinge epistemology may vary concerns the scope of hinges. Some views take a contextualist approach, on which what counts as a hinge can vary across contexts of inquiry. For instance, “the Earth is many years old” may count as a hinge for historical inquiry, since holding it is a necessary presupposition for acquiring warrant for more specific historical claims; but this may not be a hinge for, e.g., perceptual belief (Coliva, 2015; Wright, 2004). Another avenue towards a contextualist approach is to categorize groups of hinges according to their content; for instance, following the range of examples found in Wittgenstein, Martin Kusch distinguishes between ten different kinds of hinge, including hinges for perceptual beliefs about close familiar medium-size objects and hinges for memory beliefs of salient features of one’s autobiography (2016b: 133–134; 2016a). Yet another way to formulate contextualism about hinges holds that the very same sentence might express a hinge in one context but not in another; for instance, Wittgenstein provides the example of “I have two hands” as expressing a hinge in ordinary circumstances, but not in extraordinary circumstances, such as waking up in a hospital covered in bandages, where one might then genuinely doubt whether one has hands (*OC* §23).

Pritchard (2016) takes an approach that is at once invariant but also flexible concerning the specific content of some hinges; he distinguishes between “personal” hinge commitments, such as my commitment to the proposition that my name is such-and-such; “anti-skeptical” hinges held towards the denial of skeptical hypotheses, such as my commitment to the denial of the proposition that I am a brain-in-a-vat; and the “uber” hinge that all epistemic agents share, which is a commitment to the proposition that one is not radically and fundamentally in error in one’s believing. Pritchard (2022, 33) clarifies however that despite the variety in personal and anti-skeptical hinges—personal hinge commitments for instance can vary from person to person—there is really only *one* hinge commitment: the uber hinge, of which personal and anti-skeptical hinges are just codifications. Table 1 shows how the views considered in this section answer the four taxonomical questions set out above.

**Table 1:** Taxonomy of hinge epistemology

	Propositional	Doxastic	Epistemic	Scope
Moyal-Sharrock	No	No	No	Contextually determined by operative language game
Wright	Yes	Yes	Yes	Contextually determined by cognitive projects
Coliva	Yes	Yes	No	Inescapable commitments for human cognition
Pritchard	Yes	No	No	Invariant but codified in various personal and anti-skeptical hinges
RR	Yes	Yes^a^	No	Relative to epistemic perspective

^a^As discussed above, the classification of RR as doxastic depends on what one means by “doxastic.” RR is in accord with Pritchard’s non-belief version of hinge epistemology, insofar as both accept that hinges are not rationally grounded knowledge-apt beliefs. But I construe RR as doxastic because there is reason to prefer a different, less-demanding account of belief in general than rationally grounded, knowledge-apt belief

### Hinges and relativism

Many have commented on the relativist leanings of hinge epistemology (e.g., Baghramian and Coliva 2020; Boghossian 2006; Kusch 2016; Pedersen 2022; Piedrahita 2021). While some advocates of hinge epistemology have argued that the view does not entail relativism (Coliva 2015; Pritchard 2010), others have advocated for explicitly relativist readings of hinge epistemology (Ashton 2019b; Kusch 2016). In this paper, I take no stance on whether hinge epistemology as such *entails* or is *committed to* epistemic relativism. My aim is not to reject arguments aiming to show that it is possible to defend non-relativistic versions of hinge epistemology. Instead, my task is to articulate an explicitly relativistic version of hinge epistemology and show that contrary to what is often assumed, the relativist aspect is not necessarily a problem, but can rather bring explanatory payoffs, in particular in social epistemology (see Sect. 3).

The core epistemic relativist idea is that whether an epistemic agent *S* is justified or warranted in believing that *p* is relative to, in the sense of depending upon, *S*’s epistemic framework, principles, set of assumptions, etc.—her epistemic *system*.^4^ Epistemic relativism can be understood as a view composed of the following theses: *dependence*, *plurality*, *incommensurability*, and *non-neutrality*.

*Dependence*: A belief has an epistemic status (as justified or unjustified) only relative to an epistemic system.

Dependence is essential to any construal of relativism. But in addition, relativism must be understood to include the claim that there are or that there could be more than one such epistemic system; otherwise, there would be little content to the idea that justification is relative to the one and only epistemic system available. Thus,

*Plurality*: There are, or could have been, more than one such system.

It is also a necessary feature of a genuinely relativist view that the multiple possible or actual epistemic systems are in some way distinct and incompatible with each other—a mere plurality of systems on which justification depends has no relativistic consequences if it turned out possible to translate (or collapse) epistemic systems into one overarching system, with the result that there is no real disagreement between epistemic systems. Thus,

*Incommensurability*: There are distinct epistemic systems or practices which are incompatible with each other.

Incommensurability also serves to distinguish relativism from contextualist and pluralist approaches. Contextualist approaches to hinges can acknowledge that hinges are relative to some context and that there could be multiple contexts and hence multiple hinges. But there is no inherent incompatibility between hinges across contexts. For instance, that my sense organs are functioning properly might be a hinge for perceptual knowledge in Wright’s sense; I can rationally trust this proposition when engaged in the cognitive project of forming beliefs about the world based on perception. In other contexts—say, when having my vision tested by an eye doctor, the proposition that my sense organs are functioning properly is not a hinge, while other propositions might qualify as hinges instead—for instance, the proposition that the testimony of eye doctors is reliable with respect to the functioning of my eyes. But there is no conflict between these contexts; once the context is fixed (by settling on the relevant cognitive project), there is a single set of hinges that need to be in place. Context fixes the appropriate hinges, so that there could be no rational or faultless disagreement between epistemic agents with regard to hinges, once the context is fixed.

Finally, it is crucial to the relativist view that there is no epistemically neutral “view from nowhere,” or an objective stance from which different systems can be epistemically evaluated. The existence of an objective stance for ranking epistemic systems would itself comprise an epistemic system that subsumes all others.

*Non-neutrality*: There is no system-neutral way of epistemically evaluating different epistemic systems.

Non-neutrality is inherent to the idea that all epistemic justification depends on an epistemic system (*dependence*). Any attempt to epistemically evaluate an epistemic system must itself already take place within an epistemic system; thus, there could be no system-neutral way of engaging in epistemic evaluation.

While non-neutrality is closely related to the idea that all epistemic systems are “equally valid,” equal validity turns out not to be the most plausible way to render the relativist view. To say that all systems are equally valid seems tantamount to claiming that there *is* some neutral epistemic starting point from which various systems could be evaluated—it is just that such an evaluation would yield the verdict that all the systems come out equal. In order for equal validity to be true, it would have to be the case that either (i) there is a neutral stance that is itself be free of any epistemic system, contradicting the dependence claim, or (ii) there would have to be a neutral epistemic system that could translate and rank all epistemic systems, so that they could turn out as “equally valid,” contradicting incommensurability. The equal validity claim, often attributed to epistemic relativism, is actually in tension with it (see Ashton 2019b for discussion of this point). I shall therefore construe epistemic relativism in terms of *non-neutrality* rather than *equal validity*.

Epistemic relativism seems also to support a claim of *groundlessness* (Kusch 2016). According to relativism, the epistemic justification for my beliefs depends on my epistemic system and that epistemic system is only one among others, none of which enjoys a privileged objective foundation with respect to the others—thus, the justification for my beliefs seems groundless in lacking objective foundations. This groundlessness may seem to be epistemically problematic insofar as it encourages skepticism—how can we have knowledge if our beliefs are all rooted in epistemic systems that lack objective foundations? However, hinge epistemology shows how this kind of groundlessness in fact does not have skeptical results, as I discuss in Sect. 2. Hinge epistemology provides a dissolution to the problem of radical skepticism that also addresses the concern that epistemic relativism leads to a skeptical result.

It is common ground that hinge epistemology satisfies *dependence* and *groundlessness*—these are just other ways of spelling out the two core commitments of hinge epistemology, *legitimacy* and *lack of evidential support*. In order to assess whether hinge epistemology is committed to epistemic relativism, we must consider whether it is also committed to *plurality*, *incommensurability*, and *non-neutrality*. Here, there is room for debate, both about whether Wittgenstein’s own ideas are committed to relativism and whether contemporary developments of hinge epistemology are or must be. For instance, Coliva and Pritchard each seem to disagree with the *plurality* claim, arguing that there could not be multiple epistemic systems with distinct and non-overlapping hinge commitments. Coliva, for instance, argues that it is not possible for beings like us (i.e., human beings) to form beliefs about the external world using a different system of basic assumptions than we actually do (2015). Pritchard argues that despite the possible diversity among personal hinge commitments, on his view, “there is really only one overarching hinges commitment–viz., the *über hinge commitment*” (2016, 33), a commitment everyone shares to the proposition that one is not radically and fundamentally mistaken in one’s beliefs (2016, 95) and that there will be sufficient overlap in personal hinge commitments to attribute a shared epistemic system (2018). Again, I do not take a stance on whether hinge epistemology generally is committed to relativism, in exploring the viability and explanatory benefits of an explicitly epistemic relativist version of hinge epistemology.

### RR and hinges in epistemic perspectives

In this section, I set out an account of hinge commitments on which they are relative to individual epistemic perspectives. I provide an argument for this account in Sect. 2; here, the goal is simply to lay out the main tenets.

RR makes two central claims: one concerning the characterization of a “hinge commitment,” the other what I take to be a consequence of this characterization (explored in more detail in the next section), concerning the rational status of hinge commitments:

i) Hinge commitment: A hinge commitment is a propositional attitude of maximal subjective certainty towards the truth of some proposition, where that commitment is partly constitutive of an epistemic perspective.
ii) Non-evidential status: Hinge commitments are neither directly rationally supported by nor directly rationally supportive of other doxastic attitudes and are thus arationally held.

What makes a hinge a hinge, according to RR, does not have to do with features of the *content* of certain propositions and how that content relates to the justification for propositions with related content, but the *role* the commitment plays within an individual’s epistemic perspective, namely, that hinges play a constitutive role in shaping an agent’s manner of engaging with evidence. In contrast to Wright’s (2004) and Coliva’s (2015) accounts, hinges are not understood here as the necessary presuppositions of some central epistemic practice, in the way that “There is an external world” is supposed to be a necessary presupposition for the acquisition of warrant for perceptual beliefs. It does not matter so much *what* one is committed to, but *how* one is committed to it which determines whether some commitment is a hinge in an epistemic perspective.

According to RR, hinges are relative to individuals’ epistemic perspectives. Following Elisabeth Camp’s influential work on perspectives (2019), we can understand a perspective as an “open-ended disposition to … encounter, interpret, and respond to some parts of the world in certain ways” (2019, 24). An *epistemic* perspective, by extension, is an open-ended disposition to encounter, interpret, and respond to *evidence* in certain ways. Epistemic perspectives are closely related to epistemic *styles*, or ways “of interacting with evidence that expresses (aspects of) a unified set of epistemic parameters” (Flores 2021, 8), including parameters such as preferences for collecting true beliefs versus avoiding false ones, weightings of theoretical values, and policies for allocating trust in other agents, among other things (Flores 2021, 9). One difference is that where epistemic styles shift across contexts (Flores 2021, 11), an agent’s own epistemic perspective is relatively stable across contexts (Camp 2019, 10). Epistemic perspectives comprise general and unified ways of seeking and engaging with evidence.

Although perspectives themselves are not propositionally structured, epistemic perspectives can be “essentially connected” with certain propositions, in that it is partly constitutive of the perspective that it involves commitment to a certain proposition. For instance, the epistemic perspective of an evangelical protestant may essentially involve commitment to the proposition that the bible is the word of God (Camp 2019, 25). A commitment to that proposition infuses the epistemic evaluation of individuals with that perspective, in at least two ways. First, this commitment rules out hypotheses incompatible with that commitment as epistemically irrelevant, such as the hypothesis that the bible could be fallible as a guide to knowledge. This sets constraints on what evidence is available to a person with this perspective. While it is contingent that an individual has some particular epistemic perspective rather than another and thus contingent *which* constraints that individual works with, it is not contingent that, as an epistemic agent, there will be *some* epistemic constraints of this sort. That is, having commitments that constrain what can count as evidence for a perspective is not a contingent limitation of epistemic perspectives, but is essential to them, and essential to epistemic agency.

Second, this commitment frames how individuals interact with evidence in a unified way. For instance, this commitment could inform an approach to evidence that closes off inquiries once they are taken to be settled by biblical interpretation, in contrast to more skeptical styles that would leave conclusions subject to revision in light of further evidence of a different (non-biblical) kind—that is, the commitment provides an “off-ramp” to inquiry that allows certain questions to be stably settled. By contrast, consider an alternative scientific epistemic perspective, one characterized by the commitment to the scientific method as the most reliable guide to knowledge generally. Such a commitment also rules out certain hypotheses as epistemically irrelevant—such as the hypothesis that some events occur by supernatural means—and also frames interaction with evidence in a unified way, for instance, by giving greater weight to methods such as induction and logical inference than to methods such as testimony from authority as a means to acquiring knowledge (cf. Lynch 2016, 251). In sum, the core commitments of epistemic perspectives guide inquiry in two ways: they constrain inquiry (1) by ruling out certain hypotheses from investigation and (2) by facilitating certain modes or styles of epistemic engagement.

Hinge commitments, as constituents of epistemic perspectives, are manifested in the dispositions of individuals to interact with evidence in certain unified ways. In this approach, information does not provide evidence to an epistemic agent in isolation, but always only relative to an epistemic perspective. For information *i* to be able to provide evidence *E* in a way that could be appreciated as such by an epistemic agent *A*, *A* must have an appropriate epistemic perspective to facilitate being able to take *i* as evidence. To illustrate, we can consider the following example from Wittgenstein (*OC* §461):

Suppose that I were the doctor and a patient came to me, showed me his hand and said: ‘This thing that looks like a hand isn’t just a superb imitation–it really is a hand’ and went on to talk about his injury–should I really take this as a piece of information, even though a superfluous one? Shouldn’t I be more likely to consider it nonsense, which admittedly did have the form of a piece of information? For, I should say, if this information were really meaningful, how can he be certain of what he says? The background is lacking for it to be information.

In this case, the hinge commitment that one has hands prevents the claim that “this really is a hand and not an imitation” from being able to operate as information for Wittgenstein in his imagined role as physician. The case of course uses an example that is very likely to be a hinge for Wittgenstein and his readers. To highlight how the status of information as possible evidence is dependent on epistemic perspective, consider another of Wittgenstein’s examples, that of a community which believes that people were taken to the moon in their sleep (*OC* §667). Wittgenstein imagines a scenario where he comes into contact with such a community—in such a case, Wittgenstein himself of course does not regard it as possible to get to the moon, much less to do so in one’s sleep. Yet this community does regard it as possible. The take-away is that it would be possible for members of such a community to regard a person as mistaken about whether they have been to the moon, whereas Wittgenstein could not regard himself as possibly mistaken about this. The same piece of information—remembering having had a dream—would be regarded as evidence for different things; in particular, for this hypothetical community, the dream memory could be seen as evidence of having been to the moon, while for Wittgenstein, it could not.

## Certainty, rational grounding, skepticism

In this section, I provide an argument for the existence of hinges within individual epistemic perspectives. The argument simultaneously provides a dissolution of certain skeptical problems (with a parallel structure to that offered in Pritchard 2016). One of the aims is to show that an epistemic relativist version of hinge epistemology can address skeptical problems in the same manner as non-relativist versions. This is an important result in its own right, since a common concern about relativism is that it can lead to skeptical results.

### The rational grounding principle

A core piece of the argument is a principle concerning when an agent can have rational grounds to believe or doubt. We can find articulations of this principle in Wittgenstein’s writings and in some approaches to hinge epistemology.

*The rational grounding principle*: For *S* to have rational grounds *g* to doubt (or believe) that *p*, *g* must themselves be initially more subjectively certain for *S* than the proposition *p* to be doubted (or believed) is for *S*.

The guiding idea behind this principle is that the degree of certainty that we have with respect to our grounds transmits to the attitude we hold towards that for which they are grounds. For instance, something seems to have gone wrong epistemically if I am very confident in my belief that it will rain tomorrow, but on the grounds of testimony from my neighbor whom I know from experience to be an unreliable source concerning the weather. While my neighbor’s testimony could provide me with *some* grounds to believe it will rain tomorrow if I did not already believe this, it would be epistemically inappropriate to be *more* certain that it will rain purely on the basis of testimony than I am in the reliability of the testimony. To be strongly confident of something on the basis of unreliable testimony would rather seem to exhibit gullibility. The rational grounding principle offers an appealing explanation of what is wrong in this situation: my grounds for belief are not as certain to me as my belief for which they are to be grounds, and it is epistemically inappropriate for less certain grounds to ground a more certain belief.

Some of Wittgenstein’s comments in *OC* suggest endorsement of the rational grounding principle. For instance, in a passage where he considers the conditions under which one could have grounds for doubt, Wittgenstein writes (§125):

If a blind man were to ask me, ‘Have you got two hands?’ I should not make sure by looking. If I were to have any doubt of it, I don’t know why I should trust my eyes. For why shouldn’t I test my eyes by looking to find out whether I see my two hands? What is to be tested by what? (Who decides what stands fast?)

The reason Wittgenstein could not make sure he has hands by looking is that the reliability of his eyesight is not more certain to him than that he has hands. The suggestion is that any reason to doubt that he has hands would equally provide reason to doubt the reliability of his eyesight, since that is just as certain to him. The general answer to “what is to be tested by what?” that we can extract from this is that what is initially *less* certain is to be tested by what is initially *more* certain.

The rational grounding principle appears to figure as a central aspect of Pritchard’s version of hinge epistemology (see Pritchard 2016, 65, which also quotes §125 of *OC*). Below, I sketch a Pritchard-inspired argument based on the rational grounding principle. Before that, I need to address some skepticism about the rational grounding principle itself. Although *prima facie* plausible, Matt Jope has argued that the rational grounding principle has problematic consequences and so should be rejected (2019). Jope also argues that Pritchard’s hinge epistemology crucially relies on this principle, and so these problems also undermine Pritchard’s hinge epistemology. In response, Pritchard has argued that in fact, neither Wittgenstein nor himself are committed to the rational grounding principle, though it is a “reasonable misunderstanding” to think that they are (2021, 3659). I take a different approach—since my argument does endorse the rational grounding principle, it is important for me to address Jope’s worry directly.

Jope argues that the rational grounding principle clashes with a basic principle of rationality. In particular, the rational grounding principle appears to demand that when engaging in deductive reasoning, we should be more certain of the antecedent of a conditional than we are of its consequent. Yet having the doxastic state of being more certain of the antecedent than the consequent of a conditional is irrational. For example, (R) that *this is a red ball* entails that (B) *this is a ball*; R → B. In order for me to come to believe B on the basis of believing R and believing R → B, the rational grounding principle demands that I initially be more certain that R and that R → B, than I am that B. But that would be irrational, because given that R entails B, it cannot be the case that R is more certain than B. (It cannot be more probable that this is a red ball than it is that this is a ball.) As Jope puts it (2019, 3572):

To be more certain that p than that q is to believe that one possible state of affairs is that p and not-q. At the same time, to believe that p deductively entails q is to believe that it is not possible that p and not-q ... Therefore, to be more certain of a proposition p than of something one believes to be a deductive consequence of p [as apparently required by the Rational Grounding Principle], is to believe a contradiction, namely: it is possible that [p&not-q] and it is not possible that [p&not-q]

This critique misses the mark against the rational grounding principle as specified above, and seeing why this matters helps to illustrate the structure of RR. Jope’s argument assumes a static picture of rational grounding, whereas the rational grounding principle is inherently dynamic, as it describes a requirement on epistemically permissible *transitions* between degrees of subjective certainty. The grounds for belief (or doubt) are to be *initially* more certain to the epistemic agent than that which *is to be* believed or doubted. Initially, one can be more certain that p than that q. Subsequently coming to realize or bring to awareness that p deductively entails q will then raise one’s certainty in q to the level of certainty one has in p and that p entails q.^5^ Thus, considered diachronically, the rational grounding principle does not have the consequence Jope imagines: *S* is not required at any particular time to irrationally hold the doxastic state of being more certain of the antecedent than of the consequent of the conditional.

In the rational grounding principle, “grounds” is intended to be inclusive of all the grounds that actually are to provide rational support for belief (or doubt). The rational grounds for a belief may come from multiple sources. When multiple sources constitute the rational grounds for belief (or doubt), the grounds should either each individually be more certain than what is grounded, or they should collectively have more certainty. The latter case is relevant to forming beliefs on induction. No individual observation may be more certain than the generalization to be grounded, but taken together, the conjunction of a large enough collection of observations may be more certain than what is to be grounded.

It is worth considering a potential counterexample to the rational grounding principle, since it highlights the limitations of the principle and can help to mitigate misinterpretations.^6^ Suppose I am unaware that I am in barn facade county. A local tells me that the barn-looking thing in front of me is indistinguishable from a barn facade. It seems that in this case, I have grounds to believe there is a barn in front of me, because I seem to see one. I also seem to have grounds to doubt that there is a barn in front of me, because I cannot tell real barns from barn facades. According to the objection, I am not more subjectively certain that there is a barn in front of me than I am that there is a barn facade in front of me, because I cannot distinguish barns from barn facades. Nor am I more subjectively certain that there is a barn facade in front of me than I am that there is a barn in front of me, because I do seem to see a barn. Hence, the objection contends, the rational grounding principle does not permit either believing or doubting that there is a barn in front of me based on the available grounds. This result would be counterintuitive, because I intuitively do have rational grounds both for believing that there is a barn in front of me (on the basis of seeing one) and for doubting it (by realizing I cannot distinguish barns from barn facades).

*Response*: The offered verdicts in the objection about what the subject of this case is more subjectively certain of is a psychological matter. We can stipulate that the subject has the certainties assigned. In this case, indeed the Rational Grounding Principle indeed does not permit either believing or doubting that there is a barn. But if that result seems counterintuitive, this is because the stipulations of what is more subjectively certain are psychologically unlikely. For instance, it seems plausible that a subject in this situation would continue to find it more subjectively certain that there is a barn than that she is looking at a barn facade. Yes, one cannot tell barns from barn facades. But if one is truly unaware that one is in barn facade county (as per the described case) and one is just told by a local that barns are indistinguishable from barn facades, it still seems rationally permissible to remain more subjectively certain that there is a barn than that there is a barn facade. If the barn facade comment from the local comes unexpectedly, it can be discounted as a piece of rather strange advice, or else raise other questions about the local’s psychology. While the local’s advice might slightly lower one’s certainty, making one somewhat wary of the barn-seeming perceptual experience (or perhaps more likely, of the local), that does not mean that one is no longer *more* certain that there is a barn in front of one than that there is a barn facade.

The rational grounding principle sets a constraint on initial degrees of certainty for something to count as a ground for belief or doubt. It does not set epistemic constraints on what initial assignments of subjective certainty are more rational than others. The purported counterexample rests on assumptions about which prior assignments of subjective certainty are more rational. Given that the subject’s certainties are as the objection supposes them to be, then it would indeed follow from the rational grounding principle that the subject does not have grounds for either believing or doubting that there is a barn. If this seems like a problematically counterintuitive result, I have suggested the explanation is not in the rational grounding principle, but in our assumptions about what prior assignments of certainty are reasonable.

### Dissolution of skeptical problems

One of the central attractions of hinge epistemology in general is that it offers a compelling dissolution of radical skeptical problems. In this section, I argue that RR also offers a hinge-style response to skepticism, and so shares in this attraction.

Contemporary discussions of radical skeptical problems center around the use of skeptical hypotheses to undermine ordinary claims to knowledge via epistemic closure principles. Consider a skeptical hypothesis, such as the hypothesis that one is a brain-in-a-vat hooked up to a supercomputer stimulated to have the experiences that one is currently having. Such hypotheses are designed to be impossible to rule out—any evidence one might seem to have could also be fabricated in one’s experience by the supercomputer—and so apparently it is impossible to know that they do not obtain. They are also incompatible with most of what we take ourselves to know—if I am a BIV, for instance, then I am not sitting in my office drinking coffee as I take myself to be.

Epistemic closure principles describe how epistemic properties such as justification or knowledge extend across entailment relations between propositions. According to a plausible closure principle on rationally grounded knowledge (Pritchard 2016, 23):

*Closure*_*RK*_: if *S* has rationally grounded knowledge that *p*, and rationally grounded knowledge that [if *p*, then *q*], and *S* competently infers from this knowledge that *q*, *S* can come to have rationally grounded knowledge that *q*.

The skeptical problem arises when we substitute some ordinary piece of knowledge, such as “I am in my office drinking coffee” for *p*, and the denial of a skeptical hypothesis, such as “I am not a BIV” for *q*. We have already noted that it seems impossible to have rationally grounded knowledge in the denials of skeptical hypotheses. We then seemed to be faced with the choice: either give up the plausible closure principle or else admit that we cannot have ordinary knowledge entailing the denial of skeptical hypotheses.

Pritchard develops a hinge strategy for addressing this skeptical problem while retaining closure. The core idea is that hinge commitments are not eligible for entrance into closure-style inferences. This is not to say that the closure principle has exceptions, but rather that it does not apply to hinges. Pritchard’s strategy relies on his insistence that hinges are not rationally grounded knowledge-apt beliefs: His interest is in rationally grounded knowledge, and so in *Closure*_*RK*_. Given that hinges are not rationally grounded knowledge-apt beliefs, they cannot enter into an inference operating over rationally grounded knowledge.

RR takes up a similar general strategy. Hinges are not eligible for entrance into closure-style inferences, because hinges are not rationally grounded knowledge-apt beliefs (though I prefer to say that they are nevertheless genuine beliefs). RR provides a further explanation for the inapplicability of Closure_RK_ to hinges, through appeal to the rational grounding principle. The reason that hinges are not rationally grounded knowledge-apt belief is that, in virtue of their maximal subjective certainty, they are not apt to provide rational grounds for belief or for doubt, including via inferences involving known entailments. The rational grounding principle figures centrally in the explanation for why hinges should be insulated from closure-style inference.

When a maximally subjectively certain commitment is central to an individual’s epistemic perspective, that commitment is essential to the agent’s dispositions in acquiring and engaging with evidence. Showing that the core commitments of epistemic perspectives are insulated against skeptical doubts then also shows that epistemic perspectives in general are insulated against radical skeptical arguments. Ordinary rationally grounded knowledge-apt beliefs are protected against skepticism because they are formed and maintained within epistemic systems, where the essential commitments of those systems—their hinge commitments—are insulated from skeptical doubts.

Note that this hinge epistemic dissolution of skeptical arguments does not place any in-principle constraints on the contents of hinge commitments. Thus, even when epistemic systems have different and incompatible hinge commitments, those systems nevertheless are immune to the radical skeptical problem in the same way. Any epistemic system, just in virtue of having some maximally subjectively certain commitments, is insulated against radical skeptical doubts. This is not to say that there is no possibility for widespread falsehoods or epistemic vices in epistemic systems; it is just that epistemic systems are protected against radical skeptical worries due to the insularity of hinge commitments from doubt.

What renders hinge commitments insulated against skeptical doubts according to RR is their maximal degree of subjective certainty, which, together with the rational grounding principle, means that no purported reasons for doubt could succeed in directly providing rational grounds for doubt of a hinge. This resilience of hinge commitments against purported grounds for doubt is what renders RR a radically non-evidentialist view: not only are hinges rationally groundless, it is additionally not possible to directly undermine them by providing counterevidence—even if the counterevidence is true, and the hinge itself is false. An agent with an epistemic perspective with a hinge commitment to *p* will be unable to interpret information as providing evidence against *p*; but other agents with different epistemic perspectives may be able to do so.

## Applications in social epistemology

This section motivates the RR view by showing how it can be fruitfully applied to topics in social epistemology, including deep disagreement, testimonial injustice, and hermeneutical injustice.

### Deep disagreement

Hinge epistemology is already well-established as an important theoretical resource for thinking about deep disagreement (Coliva and Palmira 2021; Hazlett 2014; Johnson 2022; Kusch 2018; Lynch 2016; Pritchard 2018; Ranalli 2020; Ranalli and Lagewaard 2022a, 2022b; Smith and Lynch 2021). Here, I argue that RR is particularly well-suited for this purpose.

It is controversial how exactly to define deep disagreement (see Ranalli 2018 for discussion), but there is agreement at least on some general characteristics that a theory of deep disagreement should capture. Deep disagreements, at least in paradigm cases, are as follows (see Ranalli and Lagewaard 2022a):

i. *Systematic*: A deep disagreement usually is a disagreement not simply over a single contested proposition *p*, but extends to disagreement over various other propositions that interlocutors offer as reasons for their view.
ii. *Persistent*: Deep disagreements generally persistent even after interlocutors have offered reasons meant to convince each other. The evidence each offers the other does not succeed in changing the other’s mind.

RR explains deep disagreements as resulting from divergent hinges. Hinges, according to RR, are core commitments of individuals’ epistemic perspectives that set constraints and guide the individual’s interactions with evidence in general (as discussed in Sect. 1). A divergence in hinge commitments between agents will then be accompanied by differences in their dispositions to interact with evidence, leading to potential disagreement in many cases. Disagreements over hinges will also be persistent, because of the maximal degree of subjective certainty to which they are held. This certainty explains why hinges are insulated against doubts; there could be no grounds for doubt which themselves are more certain than the hinge to be called into doubt (see Sect. 2). Given this insularity from doubt, the reasons offered by an interlocutor in the course of an argument, and the mere fact of discovering disagreement itself, could not provide grounds to doubt a hinge. Thus, disagreements over hinge commitments are rationally persistent.

This explanation of deep disagreement takes the *persistence* feature of deep disagreement to reflect a rational irresolvability of deep disagreement—genuine deep disagreements persist even after attempts at a direct rational resolution because they are indeed not directly rationally resolvable. Some approaches to deep disagreement regard the appearance that deep disagreements are not rationally resolvable as a *problem* of deep disagreement: an explanation is then attempted to show that these disagreements really are at least indirectly rationally resolvable even though they often do not appear to be (cf. Coliva and Palmira 2021; Pritchard 2018; Ranalli 2018). This reaction—viz., to regard apparent irresolvability as a problem—is rooted in a concern about epistemic relativism, with the thought that if individuals with different hinges could not resolve the disagreement by rational means, that would imply an incommensurability in their epistemic systems that in turn leads to relativism. It is not always explained why the resulting epistemic relativism would be problematic. Assuming that the concern is that epistemic relativism yields a skeptical result, the concern can be set aside, for we have already seen how a relativist form of hinge epistemology can address radical skeptical worries.

A possible worry for a relativist hinge approach to deep disagreement is that it might fail to explain disagreement at all. That is, one might object, if two people have different views concerning whether *p* is true, where each is justified relative to their own respective epistemic systems, where is the disagreement (Ranalli and Lagewaard 2022b)? Person *A* can say that their belief that *p* is justified relative to *A*’s epistemic framework, and person *B* can say that their belief that *not-p* is justified relative to *B*’s epistemic framework, and these claims can both be true.

*Reply*: this objection really targets epistemic subjectivism, not epistemic relativism. It is characteristic of subjectivism about a discourse that when *S* asserts that *p* in that discourse, the content of *S*’s assertion is really that [*p* is true according to some standard that *S* endorses]. Subjectivism then faces the problem of lost disagreement, for what looks like a disagreement between two individuals about whether *p* is really not a disagreement at all, since the content of what one says (viz*.*, that *p* is true according to a standard that person endorses) is compatible with the content of what the other says (viz., that *p* is not true according to the standard that this other person endorses). Relativism preserves disagreement: when *S* asserts *p* and *T* asserts *not-p*, then *S* and *T* make incompatible claims. The claim of epistemic relativism is that in this situation, *S*’s assertion that *p* can be epistemically justified relative to *S*’s epistemic system, while *T*’s assertion that *not-p* can be epistemically justified relative to *T*’s epistemic system.

One might still worry that some important sense of disagreement is lost. Although *S* and *T* make incompatible claims, once they recognize that they are each justified in their claims relative to their own epistemic system, is not the rational response to this scenario to agree to disagree and acknowledge that each of their claims have equal justificatory validity? No. Relativism only entails that *S* and *T* are equally justified relative to their respective epistemic systems if some version of *equal validity* is a necessary feature of epistemic relativism. As discussed in Sect. 1.3, however, equal validity is not a necessary feature of epistemic relativism, but rather what is necessary is a *non-neutrality* claim that there is no epistemically neutral standpoint from which to assess epistemic systems. Given *non-neutrality*, *S* and *T* are rationally permitted to disagree with the other’s assertion and to critically evaluate the other’s epistemic justification. By coming to recognize the rational groundlessness of their believing, *S* or *T* may exhibit a form of intellectual humility (Johnson 2022), but merely recognizing this groundlessness does not compel them to regard the others view as equally valid or equally justified as their own.

### Testimonial injustice

Testimonial injustice occurs where a speaker’s assertion receives less credibility by the hearer than it deserves, because of an identity prejudice of the hearer (Fricker 2007). Differences in social power can contribute to testimonial injustice; when the hearer is a member of a group with more social power than the speaker and this power differential is reflected in a prejudice (explicit or implicit) of the hearer against members of the speaker’s social group, the hearer may be inclined to treat the speaker’s testimony as less credible than she would have done in the absence of an identity prejudice.

What hinge epistemology offers for the literature on testimonial injustice is an explanation of how a particular form of testimonial injustice—testimonial quieting (Dotson 2011)—can occur without awareness by the hearer. Testimonial quieting occurs when a hearer fails to recognize the speaker as a knower and thus to give the speaker’s assertions proper uptake (this notion is related to the concept of illocutionary silencing; see, e.g., Langton 1993). Testimonial quieting need not (though it may) reflect an explicit intention of the hearer to disregard the speaker’s assertions as possible sources of knowledge.

The permissive view of hinges defended in this paper allows that individuals can have hinge commitments to any proposition, even false and morally pernicious ones. For instance, in communities with deeply embedded racist attitudes, practices, and histories, we might expect at least some individuals to have a commitment to the proposition that some other group is inferior or less morally worthy—even if such individuals would not acknowledge these commitments if they were made explicit. Because hinges are arational according to RR, such hinges would not be directly responsive to rational considerations; as we might expect, then, it will not be possible to resolve disagreement over these hinges by purely rational means. This is not to provide an “apology” for such hinges; we can and should, from our own epistemic perspectives, criticize such commitments (as is permitted by the *non-neutrality* claim). Although hinges are insulated from radical skeptical doubts according to RR, this does not mean they must be true. Epistemic perspectives can be normatively assessed (again, from within one’s own epistemic perspective) as exhibiting epistemic vices, or as predicated upon false hinges. The hinge epistemic dissolution of radical skeptical problems just means that one way epistemic perspectives cannot be undermined is merely by raising skeptical hypotheses.

Where a member of a socially powerful group has an identity prejudice against members of another group that is reflected in their hinge commitments, the testimonial evidence provided by members of that group may fail to receive proper uptake by the socially privileged hearer. Where one has a prejudicial hinge commitment, the testimony provided by a marginalized group may not be regarded *as* evidence at all, or if it is, it may be regarded as less certain than the evidence provided by the testimony of others. As a result, the testimony of members of marginalized groups can be silenced. The evidence-shaping role of hinges thus offers one explanation of some instances of testimonial silencing.^7^

### Hermeneutic injustice

In addition to *testimonial* injustice, Fricker (2007) identifies a further category of epistemic injustice: *hermeneutic* injustice. Hermeneutic injustice involves a “structural prejudice in the economy of collective hermeneutical resources” (Fricker 2007, 1), where “hermeneutical resources” are interpretive tools for making sense of one’s social experiences. The canonical example of a conceptual resource that addresses hermeneutical injustice is the concept of sexual assault. The articulation and dissemination of this concept aided in the expression of the significance and harms of experiences of sexual assault.

Since hinges determine what can count as evidence for what, they set boundaries on what kind of evidence one is in a position to appreciate. Aspects of one’s experiences are of course potential sources of evidence—but only if one’s hinges allow for that experience to be evidentially significant. In principle, then, an experience *e* can provide evidential support to believe *p* relative to the set of hinges associated with individual *S*’s epistemic perspective, but the same experience *e* might fail to provide evidence for *p* relative to the hinges associated with individual *T*’s epistemic perspective. As the example of sexual assault illustrates, the availability of certain concepts comprises one sort of hermeneutical resource. Hinge commitments comprise another sort; as one’s hinge commitments change, so can one’s abilities to interpret one’s experiences.

RR has been developed primarily as an individualistic approach to hinge epistemology. The idea has been that hinge commitments attach to individual epistemic perspectives, rather than domains of inquiry or universal features of rational evaluation. However, this individualistic approach is compatible with hinges also having important social roles (see Mion 2024 for discussion). Several of Wittgenstein’s examples for instance focus on how what one learns as a child brings with it certain hinge commitments that then shape how one interprets experience. See, e.g., *OC* §159: “As children we learn facts; e.g., that every human being has a brain, and we take them on trust”; §263: “The schoolboy believes his teachers and his school books”, and that a child does not learn at all that a mountain has existed for a long time, but swallows down this fact with the rest of what it learns (*OC* §143). We do not devise our hinge commitments entirely on our own, but have many of them passed down in the process of entering into certain language games.

Taking the social genesis and maintenance of hinge commitments together with their evidence-shaping role, we can expect that structures of oppression will have implications for which hinge commitments will have currency within communities and thereby the hermeneutic resources available for describing systematic inequities. And indeed, we can see the tools of epistemic injustice at work in educational contexts. For instance, in a 2017 study, the Southern Poverty Law Center surveyed how the history of slavery was being taught in US high schools (Schuster 2018). The survey found, among other things, that textbook content tends to center on the white experience and to neglect the lived experiences of enslaved people and often fails to connect the legacy of slavery to the present day. Though it is difficult empirically to tie the effects of this educational limitation directly to present day manifestations of anti-Black racism in the USA, RR can contribute an explanation of the mechanisms by which what is included (and omitted) in early educational materials can prevent proper uptake of evidence about racial injustice. An educational system that paints a “progressive view of American history that can acknowledge flaws only to the extent that they have been addressed and solved” (Schuster 2018) will tend to encourage a set of hinge commitments that hinder the expression and understanding of experiences of persistent structural racism. In the context of and as a result of an educational system that encourages a hinge commitment to the proposition that there has been consistent social progress in the USA, attempts to express more critical perspectives may be ineffective because the hinge prevents their uptake.

### RR as a social hinge epistemology

The applicability of RR to each of deep disagreement, testimonial injustice, and hermeneutical injustice depends upon features of RR not shared by other prominent approaches in hinge epistemology. In particular, in each application, the offered explanation depended upon the assumptions that hinges could vary substantially across individuals and that hinges are insulated against doubt, yet both of these assumptions are rejected or limited in other prominent approaches. For instance, Pritchard and Coliva reject or downplay the variability in hinges across individuals in order to avoid commitment to the *plurality* claim of epistemic relativism. In so doing, however, these views are limited in their potential for explaining deep disagreement, although both nevertheless attempt such an explanation (Coliva and Palmira 2021; Pritchard 2018).

According to Pritchard, while there can be some variability in which propositions codify the uber hinge commitment, ultimately, this variability is limited: there must be sufficient overlap in hinge commitments if we are even to be able to understand each other (2018). This approach does allow for some variation in hinges and, hence, can be applied to explain deep disagreement. Because there is necessarily a significant amount of overlap in hinges, there is a possibility that deep disagreements could be rationally resolved by *indirect* means, through appeal to shared hinge commitments, rather than directly arguing about a contested hinge. If all hinge disagreements can be rationally resolved in this indirect way, the “problem” of deep disagreement can be avoided. While this is a sensible strategy for avoiding incommensurability, it does rely upon the optimistic assumption that in all cases of hinge disagreement, an indirect rational resolution will be possible: That is, that eventually, under rationally optimal conditions, interlocutors could resolve the disagreement by finding common ground in shared hinge commitments. This seems like a contentious assumption to make: Given the persistence of actual instances of disagreement (especially in deep moral or political disagreement), it seems too optimistic to hope for the possibility of indirect rational resolution in every case. RR accepts the possibility that some disagreements may be rationally irresolvable.

Coliva rejects the possibility of variation in hinge commitments altogether (at least among us humans). In her view, hinges are inescapable for beings like us: our epistemic practices are “humanly inescapable” (Coliva and Palmira 2021, 411). This restrictive approach to hinges does clearly avoid incommensurability and plurality in hinges (among human beings) and, hence, avoids commitment to relativism. However, by the same token, Coliva’s extended rationality view is unable to explain deep disagreement in terms of divergences in hinge commitments, which is the explanation offered by RR. Instead, a “hinge disagreement” on Coliva’s view could only be a disagreement concerning whether something *is* a hinge or not—not a disagreement between incompatible (but genuine) hinges. Coliva also seems to regard all hinges as *true*—i.e., that a false proposition could not play the role of hinge commitment. (Wright also seems to assume this, though it is not clear that his view is committed to the truth of hinges—see Ranalli 2018 for discussion.) Yet for RR, the possibility of false hinges and even of morally pernicious ones is important to the explanation of forms of testimonial and hermeneutical injustice.

## Conclusion

This paper introduced a radical relativist approach to hinge epistemology. RR shares with other forms of hinge epistemology an innovative response to radical skeptical problems. RR also holds promise in application to areas in social epistemology, such as the epistemology of deep disagreement, testimonial injustice, and hermeneutic injustice. Many questions remain, however. One important set of questions concern how hinge commitments can change over time, given that they are not directly responsive to rational considerations. It is also important to consider the moral, political, and epistemic obligations we might have to work to change the hinge commitments of ourselves and others.

For instance, it is an important question, to what extent we can be held intellectually or morally responsible for our hinge commitments. One might think that because hinge commitments are beyond rational evaluation, are something over which we lack direct voluntary control, we cannot be responsible for them. One does not choose the epistemic communities into which one is born and hence which propositions one “swallows down” as hinge commitments in growing into that community (*OC* §143). However, at a certain point, we must be deemed responsible for the company we keep and the biases we do not acknowledge and counteract. One of the challenges of taking responsibility in this way is that it requires us to attempt taking up perspectives other than our own, to be able to get some critical distance on our ordinary ways of engaging with the world.

## Data Availability

We do not analyze or generate any datasets, as the work proceeds within a theoretical approach.

## Footnotes

1 To be clear, this approach is not very likely to be faithful to Wittgenstein’s own view. As Coliva, Moyal-Sharrock, and Pritchard all note, Wittgenstein’s comments do strongly suggest that he does not think of hinges as propositions. My aim here however is to construct a viable theory of hinge epistemology, one that, although inspired by Wittgenstein’s writings, is not simply a project of Wittgenstein exegesis. In light of the problems with a non-propositional approach—such as an analogue of the Frege-Geach problem for hinge commitments—the aim is to capture Wittgenstein’s insights within a more plausible framework.

2 An anonymous reviewer suggests that the minimal account is insufficient because it would count as beliefs many things that in fact are not beliefs—for instance, supposedly, *inferring* requires a strong commitment in the truth of a proposition but is not a belief. I must confess I do not share the reviewer’s opinion on the matter. If, through inferring that p, one has a strong commitment to the truth of a proposition that p, then I do not see why it would be incorrect to say one comes to have the belief that p. Moreover, I find that the rationally grounded knowledge-apt notion of belief itself faces the reverse difficulty: namely, it *excludes* from the category of belief things that are in fact beliefs. For instance, one of the central issues in the literature on basic self-knowledge (such as my knowledge that I would like a cup of tea right now) concerns the epistemic status of basic self-beliefs. Basic self-beliefs are interesting and worth study by epistemologists precisely because they are beliefs that seem to lack a genuine reflectively accessible epistemic basis (though some argue that there is some covert epistemic basis at work); it is not readily apparent to subjects what counts as a legitimate answer to the question, “how do you know that you would like a cup of tea right now?”. Some argue that these are beliefs that lack an epistemic basis and are not responsive to reasons (see, for instance, Bar-On 2004). The question under discussion in this literature is not whether there are self-beliefs at all (as one would expect using a robust conception of belief), but whether and how self-beliefs have an epistemic basis. To assume that the notion of belief of interest to epistemologists must be rationally grounded knowledge-apt belief would rule out such a view from the outset, when it should at least be a point of legitimate epistemological debate whether such a view is correct.

3 An anonymous reviewer suggests that the decision of what account of belief to adopt is not merely terminological and that hinge epistemologists should be concerned with knowledge-apt belief only, since that is the kind of belief relevant to epistemology. I aim to address issues relevant to epistemologists. But it seems counterproductive to do so by building into the notion of belief precisely the epistemic issues that are important for hinge epistemologists to address. I would prefer to rely upon the minimal account of belief and say that a subset of beliefs is knowledge-apt and a subset of knowledge-apt beliefs is rationally grounded knowledge-apt beliefs. Thus, I would prefer to say that hinges are doxastic (in a minimal sense), though this is at odds with how “doxastic” is sometimes understood in the literature on hinge epistemology.

4 The discussion of epistemic relativism below follows analyses in Kusch (2016), Ashton (2019a), Baghramian and Coliva (2020), and Piedrahita (2021).

5 To illustrate: Suppose that at *t*_0_, epistemic agent *S* has some degree of subjective certainty *C*_*n*_ in *p* and *p* → *q*, but *C*_n-j_ in *q*. At t_1_, S infers from *p* and *p* → *q* that *q*. The rational grounding principle permits this inference as rational because *S* was initially (at *t*_0_) more certain of the grounds for *q* than she was of *q*. After drawing the inference, *S*’s certainty in *q* will have increased on the strength of her certainty in the grounds so that *S* has *C*_*n*_ in *q*. (I make the simplifying assumption that the degree of certainty in the grounds transmits to the resulting degree of certainty in what is grounded and also that the maximum level of certainty that can be rationally acquired in what is grounded is limited by whatever has the least degree of certainty among the grounds.).

6 I thank an anonymous reviewer for raising this counterexample.

7 For a related analysis, see Boncampagni (2019), “Hinges, Prejudices, and Radical Doubters.”.
